# Downstream evaluation of synthetic AI-generated T_1_-weighted contrast-enhanced MR images in glioma segmentation and grading

**DOI:** 10.3389/fradi.2026.1931738

**Published:** 2026-09-23

**Authors:** Dimosthenis E. Gkotsis, Adam J. Schwarz, Jan Wolber, Lehel M. Ferenczi, Hongxu Yang

**Affiliations:** 1 Pharmaceutical Diagnostics, GE HealthCare, Athens, Greece; 2 Pharmaceutical Diagnostics, GE HealthCare, Marlborough, MA, United States; 3 Pharmaceutical Diagnostics, GE HealthCare, Chalfont Saint Giles, United Kingdom; 4 Science and Technology Organization, GE HealthCare, Budapest, Hungary; 5 Science and Technology Organization, GE HealthCare, Eindhoven, Netherlands

**Keywords:** contrast-enhanced MRI, deep learning, generative AI, glioma classification, synthetic data

## Abstract

**Objectives:**

The purpose of this study was to evaluate synthetic T_1_ weighted post-contrast MR images generated from non-contrast-enhanced MR images, using deep learning (DL) methods, for automated brain tumor segmentation and automated classification of glioma grade based on imaging characteristics.

**Materials and methods:**

Three DL models were developed for synthesizing T_1_-weighted post-contrast MRI (T_1_ce) images using a publicly available dataset: (1) a conditional neural field with shift modulation (CoNeS) model, (2) a denoising diffusion probabilistic model (DDPM), and (3) a hybrid CoNeS + DDPM model. Five experimental settings were evaluated, incorporating combinations of real or synthetic T_1_ce and pre-contrast images. Synthetic T_1_ce images were evaluated in terms of image quality metrics, accuracy of tumor segmentation, and classification of cases into high-grade vs. low-grade glioma. Two classifier families were evaluated: (a) radiomics-based machine learning classifiers using random forest on extracted radiomic features, and (b) deep learning classifiers employing a convolutional neural network.

**Results:**

The combined CoNeS + DDPM model performed best in image quality and similarity metrics on the test set. Bootstrap analysis revealed that radiomics-based ML and deep learning-based classifiers exhibited distinct characteristics, each of which outperforming the other in different metrics.

**Conclusions:**

The present study demonstrates significant advances in medical image AI synthesis by integrating stable diffusion and conditional neural fields, improving the overall quality of synthetic T_1_ce images. Nonetheless, synthetic images generated solely from pre-contrast sequences failed to consistently reproduce clinically relevant glioma features. As a result, current AI-generated T_1_ce images still lack the diagnostic fidelity required to replace true contrast-enhanced imaging in clinical practice.

## 1 Introduction

Gadolinium-based contrast agents (GBCAs) for MRI have been in use since the late 1980s, and they have now become firmly established as a key radiological option in diagnostic imaging, highlighting tissues exhibiting enhanced perfusion and permeability from those which do not, across a wide range of clinical contexts and organ systems (1).

GBCAs are generally safe, but used cautiously, especially in patients with kidney impairment, as gadolinium is eliminated through the kidneys. Rarely, some patients may have allergic reactions, but the risk is minimal compared to the diagnostic benefits provided (2). Contrast-enhanced MRI is useful in oncology for tumor detection and characterization (3), and for monitoring treatment response (4–6). It can also help distinguish between benign and malignant lesions, identify tumor boundaries, and assess tumor vascularity (7). In neurology, it aids in diagnosing non-cancerous lesions such as those of neurodegenerative disorders like multiple sclerosis (8, 9), and also infections (10, 11), and strokes (12, 13). It is also highly valuable in evaluating heart disease (14, 15), liver disease (16, 17), and joint abnormalities (18).

However, some concerns have also been raised relating to the use of GBCAs. One serious concern is nephrogenic systemic fibrosis (NSF), a rare but potentially debilitating condition primarily observed in patients with severe renal impairment. NSF involves fibrous tissue formation in the skin and internal organs, eventually leading to skin thickening and hardening, and, in severe cases, even life-threatening organ complications (19, 20). Recent studies have also raised concerns about long-term gadolinium retention in the body (21), even in individuals with normal kidney function. Gadolinium deposits have been found in the brain, bones, and other tissues, raising questions about potential long-term effects. Even though no definitive link has been established, this issue underscores the current need for exercising caution, particularly in populations requiring multiple/repeat MRI scans (22, 23). The environmental impact of gadolinium is also an emerging concern. Gadolinium is not biodegradable and can accumulate in the environment, particularly in water bodies. After medical use, gadolinium can enter the wastewater system through natural excretion. Current wastewater treatment processes are not designed to fully remove gadolinium, leading to its release into rivers, lakes, and groundwater (24, 25).

Over the past few years, significant advances in MRI have been made using artificial intelligence (AI) solutions addressing numerous aspects of the MR workflow and downstream tasks, including image reconstruction (26), acquisition acceleration (27, 28), super-resolution (29), bias correction (30), and tumor classification (31).

More recently, there have also been advances in leveraging AI methods to enhance GBCA-induced image contrast when acquired at a lower administered dose, so as to resemble images acquired at the standard dose (32–35). Another area of substantial current interest is in a task known as “style transfer”, wherein an output image is synthesized from input image(s) of intrinsically different contrast, for example the generation of a pseudo-T_1_-weighted image from a T_2_-weighted input image (36, 37), affording a reduction in scan time and overall clinical workload.

Another example, and the subject of the present work, is the synthesis of synthetic GBCA-enhanced T_1_-weighted (T_1_ce) images from a set of non-contrast-enhanced MR images. There have been several such studies published in recent years, evaluating algorithms in terms of conventional (and widely used) similarity metrics such as peak signal-to-noise ratio (PSNR) and structural similarity index measure (SSIM), and/or using qualitative image evaluations by expert radiologists (34, 38, 39).

T_1_ce images are important in the management of brain tumors, including clinical diagnosis, treatment planning for radiotherapy and stereotactic radiosurgery (40), differentiating between tumor recurrence and radiation necrosis (41), and estimating the maximal safe resection margins in neurosurgery—an important factor associated with improved progression-free and overall survival (42). Therefore, the use of synthetic T_1_ce images for consequential clinical decision-making requires rigorous assessment of their effectiveness in relevant image-related tasks. Specifically, hallucinated enhancement patterns, and missed small enhancing lesions are key aspects that would negatively impact real-world decision making, including tumor grading, treatment planning, and assessment of recurrence, and as such, evaluation of synthetic contrast-enhanced MRI should be able to probe at these key aspects downstream of image synthesis and generation.

In the work presented herein, we evaluate three algorithmic AI approaches for generating synthetic post-contrast T_1_ images (T_1_ce) from non-contrast-enhanced MR image inputs, and assess the results in terms of image quality. We also assess the performance of the common downstream image processing tasks of brain tumor segmentation (whole tumor, enhancing tumor, and necrotic core), and differentiation of high-grade from low-grade gliomas using either machine learning classifiers based on radiomic features, or deep learning classifiers.

## 2 Materials and methods

### 2.1 Computational infrastructure and data

The dataset utilized herein has been described extensively in the literature (43–46), and is known as the Brain Tumor Segmentation 2019 (BraTS19) training dataset, comprising 335 subjects with either a low-grade glioma (LGG, 76 cases) or a high-grade glioma (HGG, 259 cases), along with corresponding segmentation masks for the following tumor habitats: tumor core, enhancing tumor, edema, and whole tumor (i.e., the sum of all habitats). These masks were used as reference ground truth volumes of interest (VOIs) for evaluation of synthetic T_1_ce image quality and *de novo* segmentation of synthetic T_1_ce images.

To carry out the experiments and analysis, a five-fold cross-validation (5-fold CV) strategy was employed on the complete dataset of 335 cases, comprising 259 high-grade glioma (HGG) and 76 low-grade glioma (LGG) cases. The dataset was randomly partitioned into five approximately equal folds while preserving the class distribution. Four folds were used for training and the remaining fold was used for testing, with each fold serving exactly once as the test set and four times as part of the training set.

The experiments were conducted on an NVIDIA DGX system equipped with an NVIDIA H200 GPU (140 GB memory). The implementation was based on PyTorch (47) and the MONAI (48) framework for deep learning acceleration and medical image analysis.

### 2.2 AI models for synthetic T_1_ce image generation

Three different models for the synthesis of T_1_ce images from pre-contrast images were considered. The first was a state-of-the-art Conditional Neural Field [CoNeS (49)], which encodes positional information as a condition for image generation. The second was another state-of-the-art model, the Denoising Diffusion Probabilistic Model [DDPM (50)]. Specifically, this model is conditioned on non-contrast MR sequences (i.e., T_1_, T_2_, and T_2_ FLAIR) provided in the input channels, along with additional noisy inputs. These are then jointly used to guide the generation of the synthetic contrast-enhanced T_1_ce images. The third model involved an incremental experiment that used the CoNeS-generated T_1_ce images as an additional conditional channel for the DDPM (referred to as CoNeS + DDPM), with the expectation that the DDPM would further leverage the random Gaussian noise to provide additional information when synthesizing the T_1_ce.

All three models used the T_1_, T_2_, and T_2_-FLAIR images as conditional inputs for predicting and synthesizing the corresponding T_1_ce images, and the CoNeS + DDPM model also used the CoNeS output as an extra conditional guide. The training details of the above models can be summarized as follows:

1. The CoNeS model was trained with the Adam optimizer for 500 epochs, using a batch size of 16. The initial learning rate was set to 0.0002. The inference model was selected from the final training epoch (49).
2. The DiffusionUnet was trained to generate synthetic T_1_ce images utilizing three levels of multi-scale features with channel sizes of (128,256,256) and one residual block per scale. It was also trained using the Adam optimizer, with a learning rate of 2.5 × 10^−5^. The mini-batch size was set to 16 and was trained for 100 epochs. The inference model was selected from the final training epoch. Additionally, the DiffusionUnet model was trained for 1,000 time-sampling steps using the standard DDPM scheduler. To accelerate the image synthesis process, a coarse-to-fine sampling strategy was employed (51). Specifically, the model performed coarse sampling five times, with a step size of 50 over 1,000 sampling steps. The 5 coarsely sampled images were then averaged before proceeding to the fine sampling stage, and during that stage, the coarsely generated T_1_ce images were treated as noisier observations and were further refined by the DiffusionUnet model over the final 20 steps of the DDPM scheduler. This resulted in an effective total of 120 sampling steps, achieving approximately 9x speedup compared to the conventional 1,000-step sampling strategy (52, 53).
3. The CoNeS-DDPM model was trained by conditioning a diffusion model on the T_1_ce predictions generated by the CoNeS model, along with additional conditions from the T_1_, T_2_, and T_2_-FLAIR images. The training and inference configurations were identical to those used for the DiffusionUnet model described above. The hybrid model was implemented using a two-stage sequential training strategy. First, the CoNeS model was trained independently to generate synthetic T_1_ce images from the pre-contrast MRI sequences. After training, the CoNeS model was frozen and used to generate synthetic T_1_ce images for all training and testing subjects. These CoNeS-generated images were then directly concatenated with the original conditioning MRI inputs (T_1_, T_2_, and T_2_-FLAIR) and supplied as an additional conditioning channel to the DDPM model. Therefore, the fusion mechanism consisted solely of input-level channel concatenation, resulting in four conditioning inputs: (T_1_, T_2_, T_2_-FLAIR, CoNeS-generated T_1_ce). No feature-level fusion, feature weighting mechanism, or attention module was employed. Furthermore, DDPM training was performed after the CoNeS model had been frozen. Consequently, gradients were not propagated back through the CoNeS model, and the overall system was not trained end-to-end.

The resulting synthetic T_1_ce images (generated by the three approaches above) were compared with the corresponding real T_1_ce images using the peak signal-to-noise ratio (PSNR), the structural similarity index measure (SSIM), and the mean aggregate error (MAE). First, these were each calculated on a single representative slice including the tumor from each case, to ensure relevance of these metrics to tumor-related features. Second, PSNR and MAE were calculated from image voxels within the ground truth BraTS19 individual segmented 3D volumes of interest for whole tumor, tumor core and enhancing tumor regions. More details regarding these metrics and their application are described in Supplementary Appendix A1.

### 2.3 AI model for the T_1_ce segmentation task

A three-dimensional (3D) ResNet-10-based UNet was employed to segment the synthetic T_1_ce images and generate 3D masks corresponding to three tissue types of interest:

- Whole tumor region: including peritumoral edema, necrotic tissue, non-enhancing tumor, and gadolinium-enhanced (GD-enhanced) tumor.
- Tumor subregions: including necrotic tissue, non-enhancing edema, and GD-enhanced tumor.
- Contrast-enhanced tumor only: focusing solely on the GD-enhanced component.

The model was trained from scratch to avoid any information leakage from publicly available pretrained weights (54), as BraTS19 is a widely used dataset in the development of foundational models. Training was performed over 300 epochs, using the Dice loss function, and optimized with the Adam optimizer. The model was trained to segment 3D patches of size 224 × 224 × 144 voxels. During inference, a sliding-window strategy was applied to average predictions at the patch boundaries (48). No dedicated validation dataset or early stopping procedure was used. Instead, the final model was then selected from the last training epoch, as no significant performance differences were observed toward the end of training cycle (i.e., the performance has already plateaued by the 200th epoch, with only ±1% for the last 100 epochs).

Using the previously described 5-fold cross-validation process, all segmentation models were trained independently for each input configuration (Pre, Real T_1_ce, CoNeS, DDPM, and CoNeS + DDPM). Specifically, a separate 3D ResNet-10 UNet model was trained and evaluated using the corresponding image set for each experimental setting. Therefore, models trained using Real T_1_ce images were not subsequently tested on synthetic T_1_ce images. This design was chosen to evaluate the effectiveness of each image configuration under matched training and testing conditions.

During training, data augmentation consisted of random spatial cropping to 224 × 224 × 144 voxels, random flipping along the *x*, *y*, and *z* axes (probability = 0.2 for each axis), random intensity scaling (factor = 0.1), and random intensity shifting (offset = 0.1). During inference, MONAI sliding-window inference was employed using a window size of 224 × 224 × 144 voxels, an overlap ratio of 0.5, and a sliding-window batch size of 1.

The tumor segmentation performance on the pre-contrast images plus the synthetic T_1_ce image for each synthetic T_1_ce model was compared with that achieved using the pre-contrast images plus the real T_1_ce image as well as the performance using pre-contrast images only. Five different input image data sets were thus evaluated:

1. Pre: The segmentation model received only pre-contrast T_1_, T_2_, and T_2_-FLAIR images as inputs, serving as the baseline for evaluating segmentation performance.
2. Real: The model received the same inputs as in Pre, with the addition of the real T_1_ce image. This setup represented the upper bound of segmentation performance.
3. CoNeS: Instead of the real T_1_ce images, the model received the synthetic T_1_ce images generated by the CoNeS (in addition to all Pre series).
4. DDPM: Same as above, but the synthetic T_1_ce images used were those generated by the DDPM model.
5. CoNeS + DDPM: Same as above, but the synthetic T_1_ce images used were those generated by the hybrid CoNeS + DDPM model.

The accuracy of the resulting tumor masks was compared to the ground truth BraTS19 masks using the Dice coefficient.

### 2.4 Classification algorithms to distinguish HGG and LGG

#### 2.4.1 Radiomics-based machine learning classifier

Using the segmented masks from the tasks described above, a set of original radiomic features was extracted and subsequently used as inputs for a machine learning classifier trained to differentiate between HGG and LGG. Specifically, eighteen distinct radiomic features were extracted from voxels within the whole tumor segmentation mask corresponding to the five segmentation inputs described in Section 2.3 (above).

The radiomic feature set comprised: elongation, flatness, major axis length, minor axis length, maximum 3D diameter, sphericity, surface area, first-order energy, first-order entropy, first-order kurtosis, first-order mean, first-order skewness, grey level co-occurrence matrix (GLCM) contrast, GLCM correlation, GLCM inverse difference moment (IDM), neighboring grey tone difference matrix (NGTDM) coarseness, NGTDM complexity, and NGTDM strength. More details regarding the radiomic features extracted and utilized herein are described in Supplementary Appendix A2.

For the radiomics-based Random Forest classifier, all 18 predefined radiomic features described in the current section were used directly as classifier inputs. No feature selection, dimensionality reduction, or feature-ranking procedure was applied. Furthermore, no hyperparameter tuning was performed. That way, even though the performances might be under-assessed, comparability across methods is preserved. A Random Forest classifier with 2,000 trees and a fixed random seed was used consistently across all experiments to ensure a fair comparison among Real T_1_ce and synthetic T_1_ce image configurations.

Importantly, identical 5-fold cross-validation partitions were used for all experiments, including image synthesis evaluation, segmentation, radiomics-based classification, and deep learning-based classification. Thus, the training and testing subjects were identical across all methods within a fold, ensuring direct comparability and preventing data leakage between experiments. This allowed the added value of T_1_ce images, and the effect of using synthetic T_1_ce instead of real T_1_ce with synthetic T_1_ce, to be evaluated for the classification task.

The classification performance was quantified in terms of sensitivity, specificity, accuracy, precision, and area under the receiver operating characteristics curve (AUC), with respect to the ground truth clinical diagnoses from the BraTS19 data set. More details can be found in Supplementary Appendix A1.

#### 2.4.2 Deep learning-based classifier

A deep learning-based classification was conducted using a simple 3D ResNet-10 model, as recommended by the top-ranking solution on the BraTS19 competition leaderboard (55), in order to reduce the risk of overfitting. The 3D ResNet-10 was trained using the Adam optimizer for 20 epochs with a learning rate of 5 × 10^−4^, optimized for binary cross-entropy loss. The learning rate was scheduled using the Cosine Annealing method (56) until the end of training.

For the deep learning-based classification, we directly utilized the synthetic T_1_ce images for classification without constraining the input to any segmentation mask or radiomic feature extraction. The classification performance was quantified in terms of sensitivity, specificity, accuracy, precision, and area under the receiver operating characteristics curve (AUC), with respect to the ground truth clinical diagnoses from the BraTS19 data set.

For the deep learning classifier, the input consisted of the entire brain volume, rather than tumor ROIs or segmentation-mask-guided crops. The model therefore learned grading features directly from the input MRI volumes in an end-to-end fashion. Training augmentation consisted of random spatial cropping (224 × 224 × 144 voxels), random flipping along all three spatial axes (*p* = 0.2), random intensity scaling (factor = 0.1), and random intensity shifting (offset = 0.1). Table 1 showcases a concise channel-mapping table for both classifier families (i.e., radiomics-based ML classifiers, and DL classifiers) for all different input conditions.

**Table 1:** Channel mapping table for the input conditions for both the radiomics-based ML classifier families, and the DL classifier families.

Condition	Radiomics feature source (within whole tumor mask)	DL input channels
Pre	T_1_ & T_2_ & T_2_-FLAIR	T_1_ & T_2_ & T_2_-FLAIR
Real	T_1_ & T_2_ & T_2_-FLAIR & Real T_1_ce	T_1_ & T_2_ & T_2_-FLAIR & Real T_1_ce
CoNeS	T_1_ & T_2_ & T_2_-FLAIR & CoNeS T_1_ce	T_1_ & T_2_ & T_2_-FLAIR & CoNeS T_1_ce
DDPM	T_1_ & T_2_ & T_2_-FLAIR & DDPM T_1_ce	T_1_ & T_2_ & T_2_-FLAIR & DDPM T_1_ce
CoNeS + DDPM	T_1_ & T_2_ & T_2_-FLAIR & CoNeS + DDPM T_1_ce	T_1_ & T_2_ & T_2_-FLAIR & CoNeS + DDPM T_1_ce

### 2.5 Statistical tests

Bootstrap analysis was conducted to evaluate the difference between radiomics-based machine learning vs. deep learning comparisons across all classification metrics, and also for directly comparing synthetic vs. real images for the AUC metric, using 10,000 subject-level bootstrap iterations, with a 95% confidence interval excluding zero being interpreted as statistically significant. Further details can be found in Supplementary Tables S1–S4. In each iteration, subjects were sampled with replacement, and classification metrics were recalculated. The differences between the two methods and associated 95% confidence intervals were derived from the empirical bootstrap distributions.

For the comparison of the DSC values for each tumor subregion, Wilcoxon tests were performed for each one of the Pre, CoNeS, DDPM, and CoNeS + DDPM vs. the corresponding Real DSC values, where all testing cases were collected and included for the statistical comparison.

## 3 Results

Representative input (including real T_1_ce) and output (synthetic T_1_ce) images for HGG and LGG cases from the BraTS19 test sample are illustrated in Figures 1–4. Figures 1, 2 show a HGG case for which there was good qualitative agreement between the real and synthetic T_1_ce results for all three AI models, with only subtle differences apparent on visual inspection. In contrast, Figure 3 shows a different HGG case, in which there are more substantive visual differences between algorithms and the real T_1_ce image. Figure 4 shows 4 LGG cases with mixed visual consistency across algorithms and with the real T_1_ce image.

**Figure 1:**
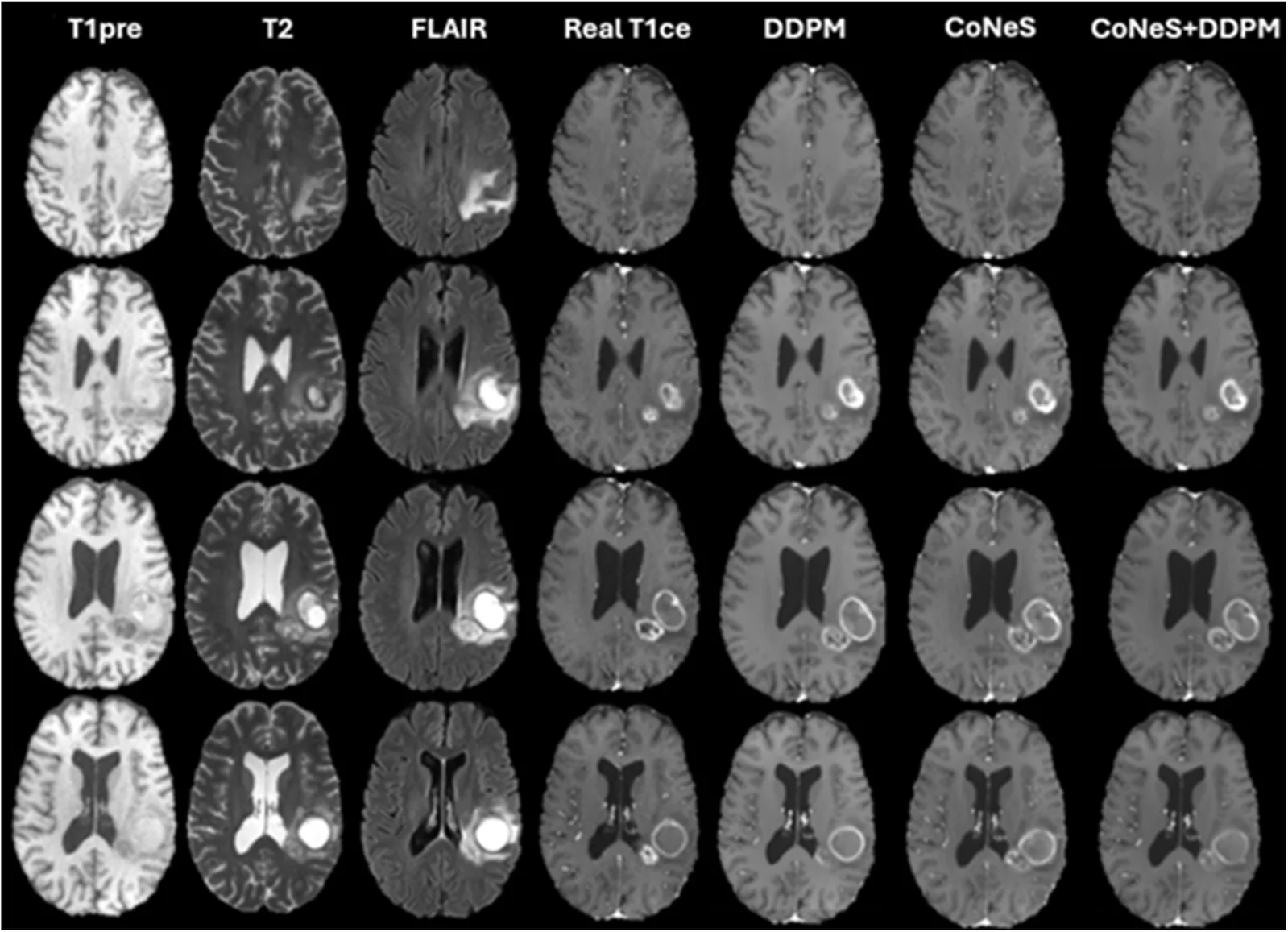
Four axial slices from a representative high-grade glioma case. The first four columns represent the input series (T_1_, T_2_, T_2_-FLAIR) and the target series (real T_1_ce). The three columns on the right represent synthetic T_1_ce images generated using the three AI methods evaluated in this study.

**Figure 2:**
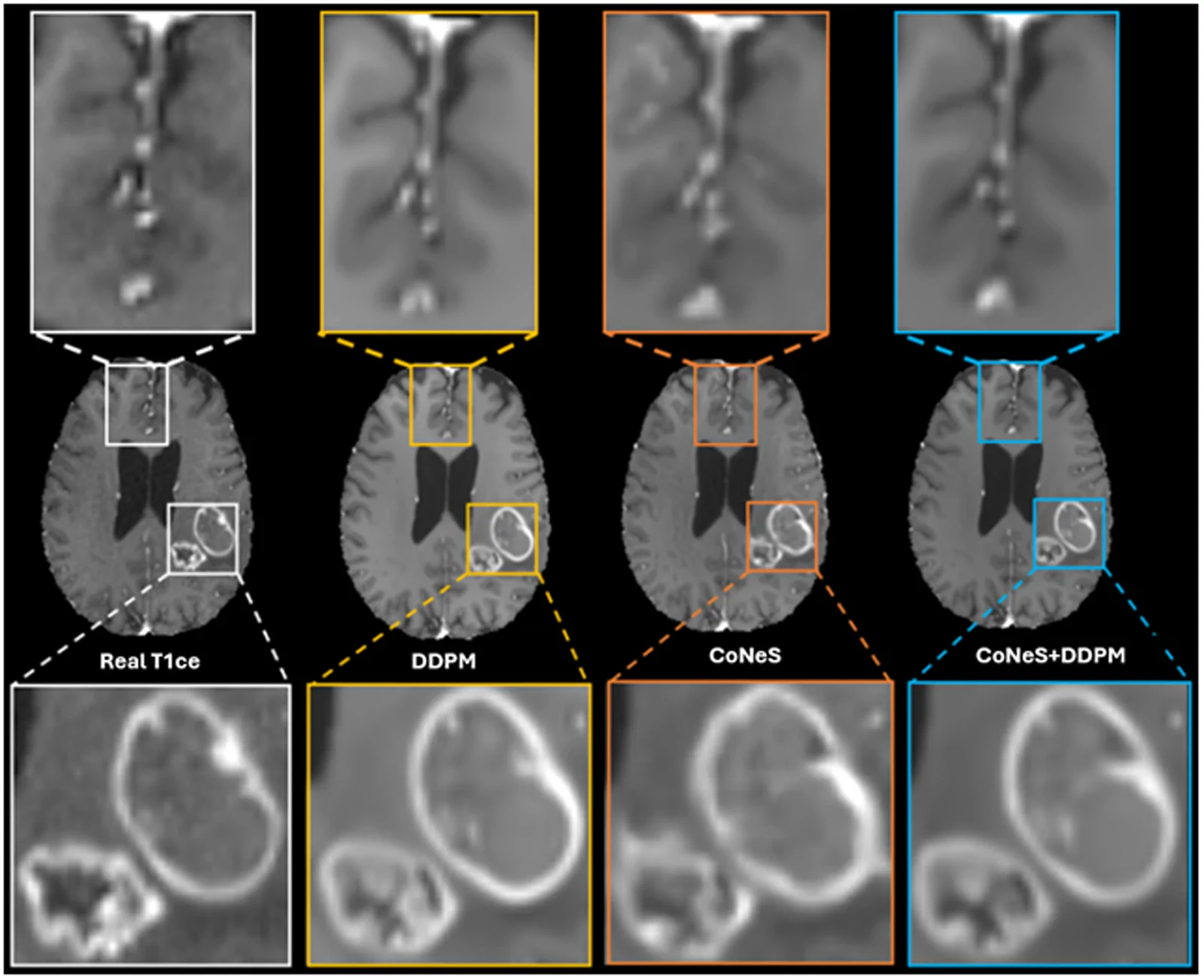
Magnified details of image features from the real (left) and synthesized T_1_ce images, from the same case shown in Figure 1, illustrating normal tissue SNR and contrast delineation (top row) and enhancing tumor (bottom row).

**Figure 3:**
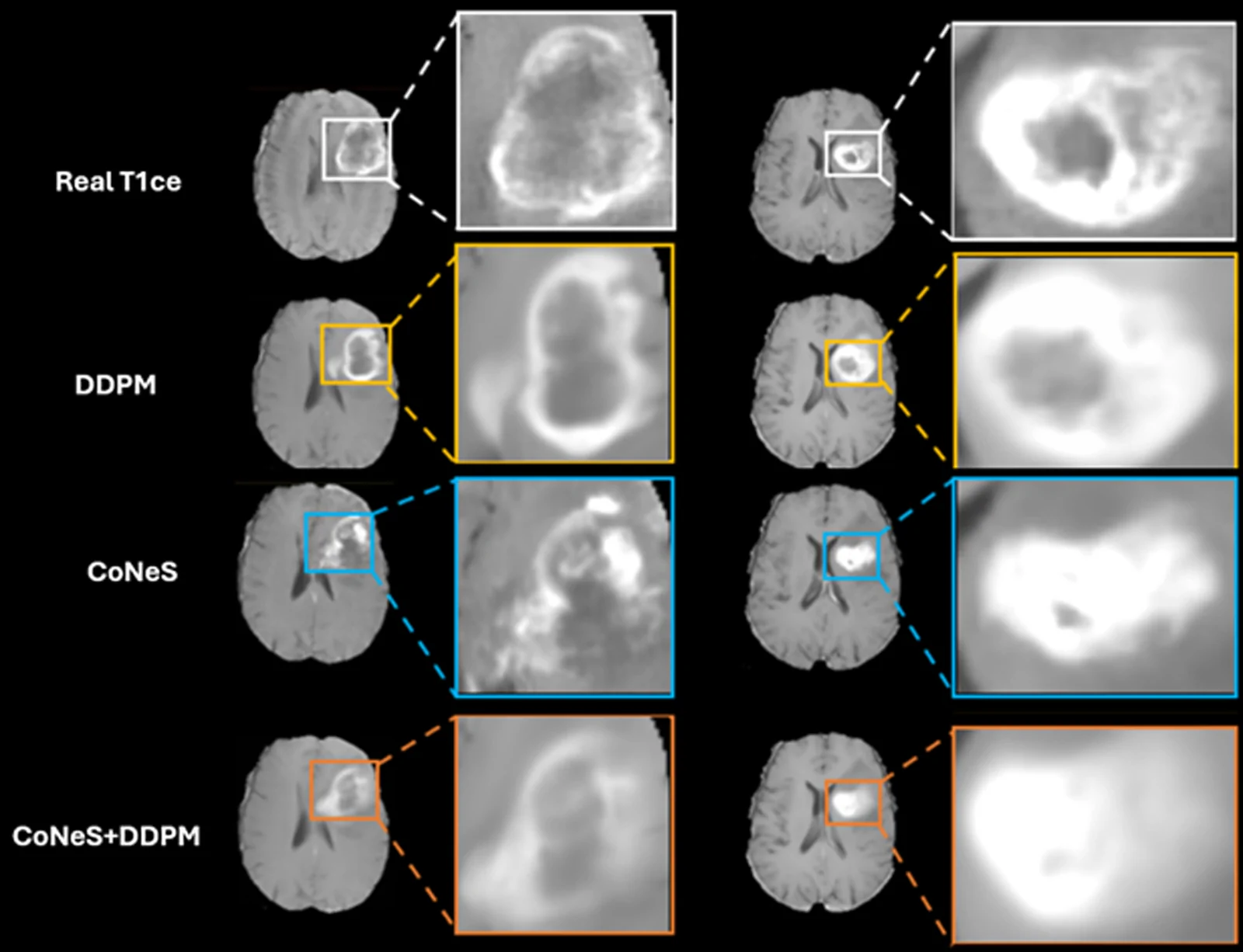
Ground truths (top row) and synthetic T_1_ce images from two additional high-grade glioma subjects (each column is one subject) for which the pattern of synthetic enhancement was more variable across the real and three different synthetic images. The tumor is shown magnified for each image.

**Figure 4:**
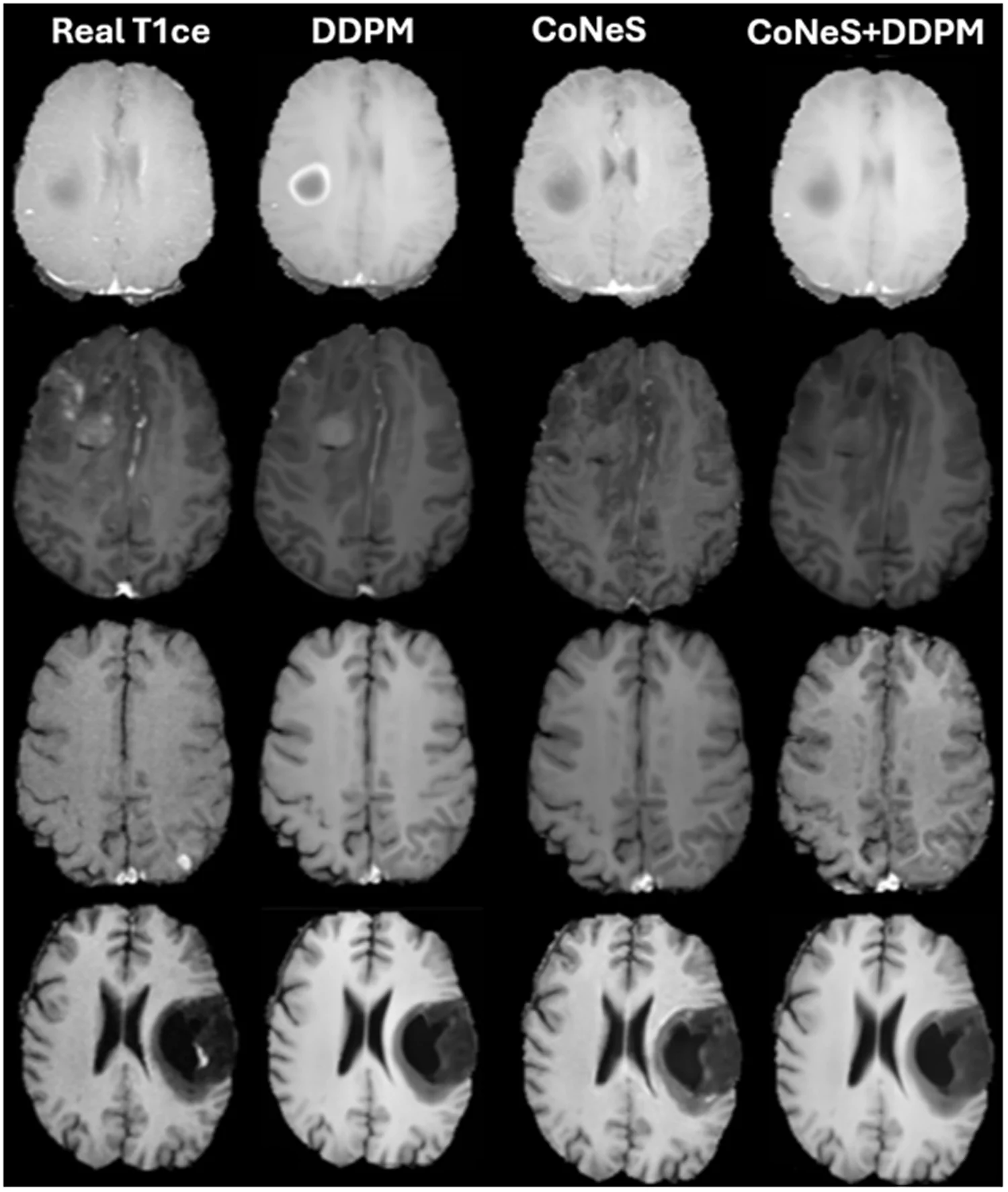
Four illustrative low-grade glioma cases from the test set. Each row corresponds to a different subject.

These examples illustrate that the degree of fidelity between purely synthetic AI-generated T_1_ce images and corresponding real, clinical standard, T_1_ce images can vary substantially across cases and with the specific nature of the AI algorithm employed. Our quantitative analysis sought to evaluate the implications of this variability on clinically relevant image segmentation and interpretation tasks across the full data set.

### 3.1 Image quality in whole slices and within segmented tumor regions

The image quality of the synthetic T_1_ce images relative to the real T_1_ce images over whole slices is summarized in Table 2. For all three metrics (PSNR, SSIM, and MAE), on the test set, the CoNeS + DDPM model numerically outperformed the other two, with the differences being most pronounced for MAE. Performance of all models was worse in the test set relative to the training set for all three metrics.

**Table 2:** Image quality evaluation of the synthetic T_1_ce images relative to the real T_1_ce images on a slice through the tumor.

Train-test split	Model	PSNR (dB)	SSIM	MAE (%)
*Training set*	CoNeS	28.66 ± 0.58	0.9651 ± 0.0021	5.10 ± 0.68
	DDPM	28.82 ± 0.30	0.9661 ± 0.0015	5.13 ± 0.33
	CoNeS + DDPM	29.47 ± 1.06	0.9654 ± 0.0059	4.59 ± 1.04
*Test set*	CoNeS	24.26 ± 0.37	0.9493 ± 0.0018	9.84 ± 0.40
	DDPM	25.10 ± 0.52	0.9545 ± 0.0017	9.40 ± 0.81
	CoNeS + DDPM	**27.27** ± **1.98**	**0.95686** ± **0.0046**	**6.81** ± **2.15**

Bold text indicates best performing algorithm for each metric and data set partition. 5-fold cross-validation mean results ± standard deviations are shown (either for the training sets or for the testing sets). Performance on the training set is provided only for illustrative purposes).

The image quality of the synthetic T_1_ce images relative to the real T_1_ce images evaluated within segmented 3D volumes of the tumors is summarized in Table 3. The best performance was observed for the whole tumor VOI relative to the core and enhancing regions (highest PSNR, and lowest MAE) for all models, while the CoNeS + DDPM model outperformed the standalone DDPM and CoNeS models for all tumor habitats/subregions.

**Table 3:** Image quality evaluation of synthetic T1ce images relative to real T1ce for regions of interest restricted to the ground truth segmented volumes.

Train-test split	Model	Whole tumor		Tumor core		Enhancing tumor	
		*PSNR (dB)*	*MAE (%)*	*PSNR (dB)*	*MAE (%)*	*PSNR (dB)*	*MAE (%)*
*Training set*	CoNeS	26.90 ± 0.70	6.79 ± 0.84	23.78 ± 0.71	11.35 ± 1.25	21.38 ± 0.62	14.70 ± 1.37
	DDPM	26.71 ± 0.31	6.75 ± 0.32	23.54 ± 0.45	11.35 ± 0.74	20.82 ± 0.60	15.53 ± 1.27
	CoNeS + DDPM	27.64 ± 0.74	6.04 ± 0.79%	24.45 ± 0.70	10.24 ± 1.04	21.79 ± 0.73	13.73 ± 1.57
*Test set*	CoNeS	21.78 ± 0.51	13.32 ± 1.06	19.10 ± 0.37	19.95 ± 0.64	17.45 ± 0.21	24.57 ± 1.11
	DDPM	22.57 ± 0.57	12.59 ± 0.97	19.85 ± 0.76	18.80 ± 1.45	17.93 ± 0.48	23.82 ± 1.30
	CoNeS + DDPM	**25.15 ± 2.34**	**8.86 ± 2.93**	**22.21 ± 2.49**	**13.87 ± 4.81**	**20.04 ± 1.91**	**17.73 ± 4.80**

Bold text indicates best performing algorithm for each metric and training or test set. Mean values from the 5-fold cross-validation ± standard deviation are shown.

### 3.2 Segmentation of tumor regions from synthetic T_1_ce images

The accuracy of segmented tumor masks relative to the ground truth BraTS19 segmentations, quantified in terms of the DSC, is summarized in Table 4. For the whole tumor target region, DSC values obtained using the synthetic T_1_ce images were ∼97%–100% of those obtained using the real T_1_ce images, across the three synthetic T_1_ce algorithms in the test set. However, results for the tumor core and the enhancing tumor tissue were worse. For the latter target region—most directly related to the rationale for using a contrast agent—the DSC values in the test set (mean value over the 5 folds plus or minus standard deviation) were only ∼58%–98% of those obtained using the real T_1_ce images, and in absolute terms ranged from 0.434 ± 0.028 (Pre model settings) to 0.739 ± 0.067 (CoNeS + DDPM model settings) relative to the ground truth masks. Finally, in Table 5, the Wilcoxon test results are shown for the paired comparisons of the DSC values of each setting (Pre, CoNeS, DDPM, CoNeS + DDPM) vs. the Real setting, indicating the *p*-values for each paired comparison, for each tumor subregion. Notably, statistically significant differences were observed for all paired assessments and all tumor subregions except from Pre vs. Real, DDPM vs. Real, and CoNeS + DDPM vs. real, for the Whole Tumor subregion's DSC values.

**Table 4:** DSC scores for the masks obtained from the segmentation of different image sets relative to the ground truth masks of the test set from the BraTS19 dataset.

Input	Dice similarity coefficient (DSC)		
	Tumor core	Whole tumor	Enhancing tumor
Pre	0.656 ± 0.034	0.875 ± 0.005	0.434 ± 0.028
Real	**0.822** **±** **0.017**	**0.878** ± **0.006**	**0.745** ± **0.022**
CoNeS	0.671 ± 0.022	0.871 ± 0.011	0.675 ± 0.027
DDPM	0.707 ± 0.029	0.874 ± 0.007	0.487 ± 0.030
CoNeS + DDPM	0.732 ± 0.065	0.876 ± 0.008	0.739 ± 0.067

Bold text indicates the best performing input image scenario for each target mask/tumor habitat. Mean values from the 5-fold cross-validation ± standard deviations are shown.

**Table 5:** Wilcoxon test results for the DSC scores for each tumor habitat, benchmarked for all 3D-resNet-10-UNet models against the real model.

Comparison	Tumor core DSC	Whole tumor DSC	Enhancing tumor DSC
Pre vs. Real	*p* < 0.05	0.977	*p* < 0.05
CoNeS vs. Real	*p* < 0.05	*p* < 0.05	*p* < 0.05
DDPM vs. Real	*p* < 0.05	0.402	*p* < 0.05
CoNeS + DDPM vs. Real	*p* < 0.05	0.217	*p* < 0.05

### 3.3 Classification task performance

The classification results are summarized in Table 6.

**Table 6:** Classification performance of the five models.

Classifier families	Model	Accuracy	AUC	Sensitivity	Specificity	Precision
ML radiomics classifiers	Pre	0.791 ± 0.031	0.826 ± 0.047	0.936 ± 0.032	0.313 ± 0.097	0.821 ± 0.047
	Real	**0.866 ± 0.047**	**0.884 ± 0.022**	**0.946 ± 0.037**	**0.601 ± 0.122**	**0.888 ± 0.044**
	CoNeS	0.839 ± 0.024	0.865 ± 0.037	0.940 ± 0.037	0.508 ± 0.089	0.866 ± 0.039
	DDPM	0.836 ± 0.052	0.860 ± 0.036	0.915 ± 0.068	0.577 ± 0.167	0.880 ± 0.057
	CoNeS + DDPM	0.818 ± 0.030	0.873 ± 0.031	0.924 ± 0.056	0.469 ± 0.076	0.854 ± 0.040
DL classifiers	Pre	0.660 ± 0.080	0.776 ± 0.041	**0.693 ± 0.172**	0.588 ± 0.292	0.863 ± 0.085
	Real	**0.690 ± 0.070**	0.822 ± 0.041	0.660 ± 0.116	0.824 ± 0.159	0.927 ± 0.065
	CoNeS	0.678 ± 0.067	0.816 ± 0.042	0.657 ± 0.160	0.789 ± 0.265	0.922 ± 0.081
	DDPM	0.687 ± 0.065	**0.824 ± 0.044**	0.692 ± 0.159	0.711 ± 0.265	0.898 ± 0.077
	CoNeS + DDPM	0.669 ± 0.078	0.807 ± 0.036	0.609 ± 0.097	**0.890 ± 0.085**	**0.948 ± 0.043**

Bold text indicates the best performing model for each metric, for each of the ML and DL classifiers. Mean values from the 5fold cross-validation ± standard deviations are shown.

For the ML classifiers trained on radiomic features, the model using real T_1_ce input performed best on all metrics. Overall, ML classifiers using synthetic T_1_ce inputs had sensitivity values in the range of 0.915 ± 0.068 (DDPM model) to 0.940 ± 0.037 (CoNeS model), specificity values in the range of 0.469 ± 0.076 (CoNeS + DDPM model) to 0.577 ± 0.167 (DDPM model) and precision values in the range of 0.854 ± 0.040 (CoNeS + DDPM) to 0.880 ± 0.057 (DDPM model).

For the DL classifiers, the performance was more variable across classification parameters and input models, with the Real T1ce model obtaining the highest Accuracy (0.690 ± 0.070), the DDPM obtaining the highest AUC (0.824 ± 0.044), the Pre model demonstrating the highest Sensitivity (0.693 ± 0.172), and the CoNeS + DDPM model demonstrating the largest Specificity and Precision values, 0.890 ± 0.085, and 0.948 ± 0.043, respectively.

Comparing the ML and DL classification results directly for matched conditions (e.g., Pre-ML vs. Pre-DL; CoNeS-ML vs. CoNeS-DL etc.), the results from the bootstrap analyses were quite informative. Specifically, two important observations emerged. First, the radiomics-based and DL-based classifiers responded differently to synthetic image variations, demonstrating that significant differences between the two classifier families across multiple performance metrics existed. Radiomics generally achieved higher accuracy, AUC, and HGG sensitivity, whereas DL classifiers exhibited higher specificity and, in several synthetic image settings, higher precision. Importantly, the magnitude and direction of these differences varied across synthesis methods, suggesting that handcrafted radiomic features may be more susceptible to synthesis-induced alterations in image appearance. In contrast, the DL classifier maintained relatively consistent characteristics across the different synthesis approaches, indicating that learned representations may be more robust to synthesis-related variations.

Second, despite observed performance fluctuations, synthetic T_1_ce images largely preserved the information required for glioma grading. Relative to pre-contrast MRI, both real and synthetic T_1_ce inputs generally improved the classification performance.

Overall, and regarding real vs. synthetic T_1_ce, relative to Pre, both Real and Synthetic T_1_ce inputs generally improved classification results. For radiomics, AUC improved significantly for Real T_1_ce, DDPM, and CoNeS + DDPM, but not for CoNeS. For DL, AUC improved significantly for Real T_1_ce and all three synthetic inputs. In the direct synthetic-vs.-real analysis, however, none of the AUC differences were statistically significant for either classifier family, because very 95% bootstrap confidence interval included zero. All relevant tables can be found in Supplementary Materials S1–S4.

## 4 Discussion

We developed three state-of-the-art generative AI models to synthesize contrast-enhanced T_1_-weighted images (T_1_ce) of the brain using only unenhanced MRI images (T_1_pre, T_2_, and T_2_-FLAIR) as inputs. In addition to evaluating the robustness of the synthetic images using standard image quality metrics (PSNR, SSIM, and MAE), we assessed the synthetic images in several “downstream” tasks more closely related to clinical utility. Specifically, we assessed the performance on multi-habitat tumor segmentation, and case classification between HGG and LGG, in both cases using the ground truth labels provided with the BraTS19 data set. For the classification task, we evaluated classifiers based on radiomic feature extraction followed by machine learning-based classification, and on deep learning-based classification using the image data directly.

In terms of quantitative image quality metrics, all three synthetic T_1_ce models exhibited performance degradation on the testing set relative to the training set. This may be suggestive of limited generalizability (especially since no external dataset validation was utilized in this study), likely due to the inherent limitations of unenhanced imaging in accurately and fully capturing the local disruption of the blood-brain barrier. While the CoNeS + DDPM combination was numerically superior for most of these evaluations, the quantitative differences between the three algorithms were relatively small. We also observed a worse performance in terms of basic image quality metrics for tumor regions relative to the full images. This may indicate that the intensity distributions of the synthetically generated tumors are somewhat shifted with respect to the real tumor regions in the real T_1_ce images. It also highlights likely limitations in the image contrast information from the non-contrast images that can be leveraged for the synthetic image generation task, even for the state-of-the-art AI models that were evaluated here.

In terms of segmentation performance, we compared the quality of the segmented volumes generated from the synthetic T_1_ce images across the three segmentation tasks. As expected, the “Enhancing Tumor” segmentation task yielded the lowest performance compared to the “Whole Tumor” and “Tumor Core” tasks. This outcome highlights the challenge of synthesizing contrast-enhancement information from strictly non-enhancing input data (57). Finally, in comparing “machine learning” vs. “deep learning” approaches for glioma grading, it was observed that the use of synthetic T1ce data was associated with numerical differences in classification performance. however, bootstrap analysis revealed no statistically significant AUC differences between Real and synthetic T1ce conditions, regardless of whether the classifier itself was ML-based or DL-based, especially when compared to the models that were trained on real T_1_ce data.

The divergent sensitivity-specificity profiles indicate that the two classifier families capture and use grading information differently. Radiomics provided stronger case-level discrimination and HGG detection at the selected decision threshold (=0.5), while DL yielded higher specificity and for selected synthetic inputs, higher precision. Therefore, the classifiers should be interpreted as operating at different decision points rather than ranked using a single threshold-dependent metric.

Bootstrap analysis revealed two key findings: first, radiomics-based and DL-based classifiers exhibited different responses to synthetic image variations, and although radiomics generally achieved higher accuracy, AUC, and sensitivity, the magnitude of these differences varied across synthesis methods, suggesting that handcrafted radiomic features are more susceptible to synthesis-induced image perturbations. In contrast, DL-based representations showed more consistent behavior across synthetic image conditions. Second, no statistically meaningful AUC differences were detected between synthetic and Real T_1_ce for either classifier family, as all 95% bootstrap confidence intervals included zero. These results indicate that synthetic T_1_ce preserves information relevant to glioma grading while highlighting the greater robustness of DL-based representations to variations introduced by different synthesis approaches.

While in many cases (especially HGG), the synthetic images appeared to be in good qualitative (visual) agreement with the ground truth real T_1_ce images, on closer visual inspection, there were often differences between them, some of which may impact clinical assessment. First, all synthetic images exhibited less noise than the real images, attributed to the intrinsic denoising properties of the generative AI models explored in this work. Lower noise levels result in a less grainy appearance of the images, which translates into a better differentiation of grey-white matter (as evidenced by the top zoomed-in boxes in Figure 1). The anatomical structures including the midline, the lateral ventricles, and the cortex appeared to be identical with the real images. When considering the extent of the lesion, the dimensions were not visibly exaggerated (neither positively nor negatively), rendering an accurate qualitative representation of the lesion. However, subtle changes were observed within the lesion (represented by, e.g., the smaller lesion on the left of the bottom box in Figure 1). Qualitatively, the overall impression is that tumor grades would not have been either upgraded or downgraded. Similar results are also illustrated in Figure 2, where the pre-contrast input images are shown alongside both the real and the synthetic T_1_ce images. The overall quality of the synthetic T_1_ce images was exceptional, as further supported by the high PSNR and SSIM scores. However, consistent with the observations in Figure 1, the precise contrast-enhancement patterns in the synthetic images did not replicate those in the real T_1_ce images. In the bottom row of Figure 2, the hybrid CoNeS + DDPM model appears to exhibit the lowest contrast (lesion-to-brain ratio) in the ring-enhancing portion of the lesion over the necrotic tumor core, compared to the two other synthesis models.

Compared to Figures 1, 2, the real T_1_ce image data shown in Figure 3 [as well as the pre-contrast image data (not explicitly shown)] exhibited relatively poorer overall image quality. As a result, the contrast-enhancement patterns in the synthetic T_1_ce images appeared more visually divergent across models and from those in the real T_1_ce images. The lesions synthesized by the CoNeS and CoNeS + DDPM models did not exhibit the characteristic ring-enhancing appearance (highlighted in the blue box on the left), a feature which is strongly associated with high-grade gliomas and an important factor in grading decisions. This illustrates a potential algorithm-dependent risk of mis-classifying some tumors if using synthetic T_1_ce images alone, which is further illustrated in Figure 4 for four different LGG subjects from the testing dataset, where the synthetic images were qualitatively different from the real T_1ce_ image to the extent they were consequently mis-classified by both the deep learning and machine learning classifiers. These examples illustrate the hallucination of a ring-enhancing pattern (a high-grade feature) around a LGG lesion by the DDPM model (top row) and a small posterior lesion in the left occipital lobe that was almost entirely missed by all models (third row). The image in the fourth row illustrates a combination of imaging characteristics including a lesion comprising a prior surgically recessed tumor, surrounding edema, and a small contrast-enhancing region near the recession border, indicative of potential tumor recurrence. Once again, while the recessed region and the edema were accurately synthesized by the AI models, and even though the overall image quality was improved by the denoising properties of the AI models, the single most important finding (i.e., the small contrast-enhancing region) based upon which the patient's treatment pathway would be tailored, was missed by all models.

Considering the statistical results from the Wilcoxon tests on the segmentation task, and the bootstrap analysis for the classification task, it can be observed that while the classification results suggest that synthetic T_1_ce preserves the relevant information for glioma grading, significant differences remain for the segmentation task, and particularly for the enhancing tumor subregion. Therefore, although synthetic T_1_ce seem to retain grading-relevant features, the current experimental evidence suggests that does not support interchangeability with real T_1_ce images.

Limitations of this study include the fact that only a single open-sourced dataset (of limited size) was used. Moreover, this dataset suffered from class imbalance, in that the minority class (i.e., LGG) was not proportionally represented in either the training or the testing set. Finally, a formal blinded image evaluation by expert radiologists was not performed, and instead the qualitative evaluation of the synthetic images was performed by experienced medical physicists for the purposes of illustration to complement the quantitative and downstream evaluations of these images. An additional intrinsic limitation was that the dataset utilized did not include genomic and histopathological information to guide image synthesis, which can be considered as a limiting factor since such information is increasingly used to guide patient management in recent years. Finally, no independent external evaluation dataset was used due to the variety of downstream tasks that was explored in this work. Particularly, a dataset containing all required sequences, with the required annotations for the synthesis, segmentation, and classification tasks was not available to us. Further studies could expand the number and types of input series to include functional image data such as susceptibility-weighted and diffusion-weighted images for the synthesis task.

## 5 Conclusions

The present study underscores significant advances in medical image AI synthesis through the integration of stable diffusion and conditional neural fields, leading to improvements in overall image quality of synthetic T_1_ce images by leveraging the intrinsic denoising and sharpening properties of these network architectures. However, despite these improvements, the synthetic images derived from the T_1_pre, T_2_, and T_2_-FLAIR images were unable to perfectly capture the presence or absence of clinically-relevant features in all glioma cases, with potential implications for patient management decisions if the synthetic images alone were to be used for radiological evaluation. Specifically, it was observed that for both true HGG and true LGG, the enhancement patterns on the synthetic T_1_ce images were not able to replace as like-for-like the true T_1_ce images, since both missed lesions were seen (e.g., present in the real T_1_ce but absent in the synthetic T_1_ce), and false enhancement patterns were introduced (e.g., hallucinated ring-enhancing pattern in a true LGG), suggesting limited interchangeability, to the extent that is allowed to be concluded by the results presented herein. This limitation suggests that, while promising, the current state of AI-generated synthetic T_1_ce images cannot yet replicate the diagnostic value of actual contrast-enhanced images with sufficient fidelity for clinical application. Training on larger and more diverse datasets with richer types of pre-contrast input series like diffusion weighted imaging and susceptibility weighted imaging, may provide improved results.

## Figures and Tables

**Figure 5:**
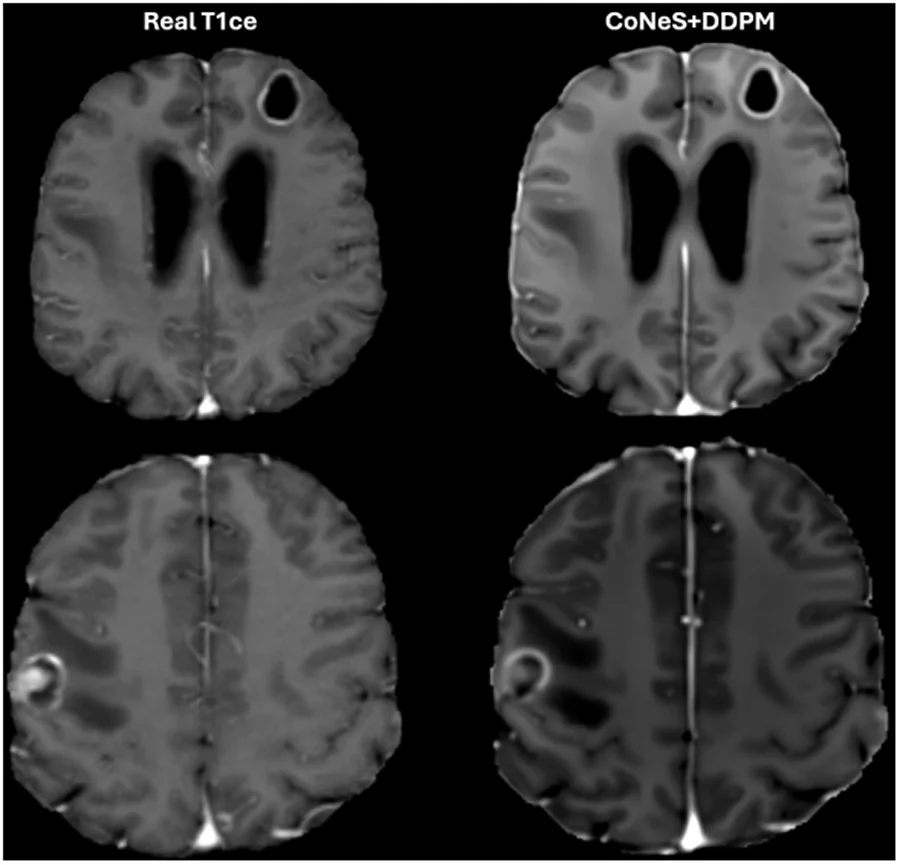
Real T_1_ce vs. CoNeS + DDPM-generated T_1_ce for two cases of high-grade ring-enhancing lesions.

## Data Availability

Publicly available datasets were analyzed in this study. This data can be found here: https://www.med.upenn.edu/cbica/brats-2019/.
