# Supplementary material for: Downstream evaluation of synthetic AI-generated T1-weighted contrast-enhanced MR images in glioma segmentation and grading
Source: Front Radiol. 2026 Sep 23;6:1931738. doi: 10.3389/fradi.2026.1931738 (PMC13642275; doi:10.3389/fradi.2026.1931738)
Supplement: Supplementary file 1 [file Supplementaryfile1.docx]

Supplementary Classification Bootstrap Tables

**Radiomics and deep learning comparisons; 10,000 subject-level bootstrap iterations**

Difference conventions: between-classifier difference = DL - Radiomics; within-classifier difference = comparator - Pre; synthetic-versus-real difference = Synthetic - Real. A 95% CI excluding zero was interpreted as statistically significant. Values displayed as p = 0.0000 correspond to p < 0.0001.

# Supplementary Table S1. Between-classifier bootstrap comparisons

| **Input** | **Metric** | **DL - Radiomics** | **95% CI** | **p-value** | **Interpretation** |
| --- | --- | --- | --- | --- | --- |
| Pre | Accuracy | -0.110 | [-0.164, -0.054] | 0.0004 | Radiomics higher |
| Real | Accuracy | -0.176 | [-0.236, -0.119] | <0.0001 | Radiomics higher |
| CoNeS | Accuracy | -0.131 | [-0.191, -0.075] | <0.0001 | Radiomics higher |
| DDPM | Accuracy | -0.149 | [-0.206, -0.096] | <0.0001 | Radiomics higher |
| CoNeS+DDPM | Accuracy | -0.149 | [-0.209, -0.093] | <0.0001 | Radiomics higher |
| Pre | Precision | -0.001 | [-0.038, 0.037] | 0.9676 | No significant difference |
| Real | Precision | +0.031 | [-0.015, 0.076] | 0.1868 | No significant difference |
| CoNeS | Precision | +0.044 | [0.001, 0.087] | 0.0458 | DL higher |
| DDPM | Precision | +0.003 | [-0.041, 0.047] | 0.8844 | No significant difference |
| CoNeS+DDPM | Precision | +0.092 | [0.050, 0.133] | 0.0002 | DL higher |
| Pre | Sensitivity | -0.181 | [-0.239, -0.124] | <0.0001 | Radiomics higher |
| Real | Sensitivity | -0.290 | [-0.351, -0.228] | <0.0001 | Radiomics higher |
| CoNeS | Sensitivity | -0.247 | [-0.309, -0.185] | <0.0001 | Radiomics higher |
| DDPM | Sensitivity | -0.228 | [-0.286, -0.174] | <0.0001 | Radiomics higher |
| CoNeS+DDPM | Sensitivity | -0.317 | [-0.382, -0.255] | <0.0001 | Radiomics higher |
| Pre | Specificity | +0.132 | [-0.013, 0.276] | 0.1066 | No significant difference |
| Real | Specificity | +0.211 | [0.066, 0.355] | 0.0086 | DL higher |
| CoNeS | Specificity | +0.263 | [0.118, 0.408] | 0.0008 | DL higher |
| DDPM | Specificity | +0.118 | [-0.026, 0.263] | 0.1406 | No significant difference |
| CoNeS+DDPM | Specificity | +0.421 | [0.289, 0.553] | <0.0001 | DL higher |
| Pre | AUC | -0.099 | [-0.175, -0.022] | 0.0124 | Radiomics higher |
| Real | AUC | -0.090 | [-0.156, -0.026] | 0.0054 | Radiomics higher |
| CoNeS | AUC | -0.060 | [-0.125, 0.006] | 0.0762 | No significant difference |
| DDPM | AUC | -0.086 | [-0.142, -0.032] | 0.0012 | Radiomics higher |
| CoNeS+DDPM | AUC | -0.082 | [-0.145, -0.019] | 0.0112 | Radiomics higher |

# Supplementary Table S2. Radiomics comparisons relative to Pre

| **Input vs Pre** | **Metric** | **Difference** | **95% CI** | **p-value** |
| --- | --- | --- | --- | --- |
| Real | Accuracy | +0.075 | [0.036, 0.113] | <0.0001 |
| Real | AUC | +0.065 | [0.019, 0.114] | 0.0056 |
| Real | Precision | +0.067 | [0.038, 0.096] | <0.0001 |
| Real | Sensitivity | +0.012 | [-0.019, 0.046] | 0.5708 |
| Real | Specificity | +0.289 | [0.158, 0.408] | <0.0001 |
| CoNeS | Accuracy | +0.018 | [-0.024, 0.060] | 0.4264 |
| CoNeS | AUC | +0.024 | [-0.026, 0.075] | 0.3636 |
| CoNeS | Precision | +0.039 | [0.011, 0.069] | 0.0080 |
| CoNeS | Sensitivity | -0.035 | [-0.073, 0.004] | 0.1002 |
| CoNeS | Specificity | +0.197 | [0.079, 0.329] | 0.0036 |
| DDPM | Accuracy | +0.045 | [0.003, 0.087] | 0.0380 |
| DDPM | AUC | +0.065 | [0.027, 0.106] | 0.0006 |
| DDPM | Precision | +0.057 | [0.027, 0.088] | <0.0001 |
| DDPM | Sensitivity | -0.019 | [-0.058, 0.019] | 0.3682 |
| DDPM | Specificity | +0.263 | [0.132, 0.395] | <0.0001 |
| CoNeS+DDPM | Accuracy | +0.027 | [-0.009, 0.063] | 0.1868 |
| CoNeS+DDPM | AUC | +0.051 | [0.010, 0.093] | 0.0152 |
| CoNeS+DDPM | Precision | +0.033 | [0.007, 0.060] | 0.0130 |
| CoNeS+DDPM | Sensitivity | -0.012 | [-0.046, 0.023] | 0.5778 |
| CoNeS+DDPM | Specificity | +0.158 | [0.039, 0.276] | 0.0120 |

# Supplementary Table S3. DL comparisons relative to Pre

| **Input vs Pre** | **Metric** | **Difference** | **95% CI** | **p-value** |
| --- | --- | --- | --- | --- |
| Real | Accuracy | +0.009 | [-0.033, 0.048] | 0.7298 |
| Real | AUC | +0.074 | [0.029, 0.119] | 0.0014 |
| Real | Precision | +0.100 | [0.064, 0.137] | <0.0001 |
| Real | Sensitivity | -0.097 | [-0.139, -0.054] | <0.0001 |
| Real | Specificity | +0.368 | [0.250, 0.487] | <0.0001 |
| CoNeS | Accuracy | -0.003 | [-0.048, 0.039] | 0.9370 |
| CoNeS | AUC | +0.062 | [0.018, 0.109] | 0.0072 |
| CoNeS | Precision | +0.084 | [0.049, 0.120] | <0.0001 |
| CoNeS | Sensitivity | -0.100 | [-0.147, -0.054] | <0.0001 |
| CoNeS | Specificity | +0.329 | [0.224, 0.447] | <0.0001 |
| DDPM | Accuracy | +0.006 | [-0.036, 0.045] | 0.8360 |
| DDPM | AUC | +0.078 | [0.033, 0.126] | 0.0008 |
| DDPM | Precision | +0.062 | [0.029, 0.096] | 0.0006 |
| DDPM | Sensitivity | -0.066 | [-0.108, -0.023] | 0.0020 |
| DDPM | Specificity | +0.250 | [0.145, 0.368] | <0.0001 |
| CoNeS+DDPM | Accuracy | -0.012 | [-0.057, 0.033] | 0.6710 |
| CoNeS+DDPM | AUC | +0.067 | [0.028, 0.109] | 0.0010 |
| CoNeS+DDPM | Precision | +0.126 | [0.086, 0.165] | <0.0001 |
| CoNeS+DDPM | Sensitivity | -0.147 | [-0.197, -0.100] | <0.0001 |
| CoNeS+DDPM | Specificity | +0.447 | [0.329, 0.566] | <0.0001 |

# Supplementary Table S4. Direct synthetic-versus-Real AUC comparisons

| **Classifier** | **Comparison** | **AUC difference** | **95% CI** |
| --- | --- | --- | --- |
| Radiomics | CoNeS - Real | -0.042 | [-0.092, 0.008] |
| Radiomics | DDPM - Real | +0.000 | [-0.039, 0.040] |
| Radiomics | CoNeS+DDPM - Real | -0.014 | [-0.050, 0.023] |
| DL | CoNeS - Real | -0.012 | [-0.038, 0.015] |
| DL | DDPM - Real | +0.004 | [-0.013, 0.022] |
| DL | CoNeS+DDPM - Real | -0.007 | [-0.045, 0.032] |

# Appendices

***A.1. Evaluation Metrics***

The following metrics were used for evaluating the synthesis models, and the various downstream models throughout this work.

*Peak Signal-to-Noise Ratio (PSNR)*

$$PSNR=10\cdot\log_{10} \left( \frac{MAX^{2}}{MSE} \right)$$

Where MAX corresponds to the maximum signal within the region under evaluation, and MSE stands for the mean squared error, and is given by: $MSE=\frac{1}{N}\sum_{i=1}^{N} \left( y_{i}- \hat{y_{i}} \right)^{2}$, with $y_{i}$ corresponding to the value of the i^th^ pixel in the image under evaluation, and the $\hat{y_{i}}$ corresponding to the i^th^ pixel in the ground truth image, against which the image under evaluation is evaluated.

*Structural Similarity Index Measure (SSIM)*

The SSIM is calculated between two different images, x and y, of common size, corresponding to two identical locations within each image, and is given by:

$$SSIM\left( x,y \right)= \frac{(2\mu_{x}\mu_{y}+c_{1})(2\sigma_{xy}+c_{2})}{(\mu_{x}^{2}+\mu_{y}^{2}+c_{1})(\sigma_{x}^{2}+\sigma_{y}^{2}+c_{2})}$$

Where $\mu_{x}$ and $\mu_{y}$ are the pixel means of x and y, respectively, $\sigma_{x}^{2}$ and $\sigma_{y}^{2}$ are the sample variances of x and y, respectively, $\sigma_{xy}$ is the sample covariance of both x and y, $c_{1}$ and $c_{2}$ are two constants meant to stabilize the division with the denominator and which are given by $c_{1}=\left( \kappa_{1}L \right)^{2}$ and $c_{2}=\left( \kappa_{2}L \right)^{2}$, respectively, with L corresponding to the dynamic range of the pixel values, and with $\kappa_{1}$ and $\kappa_{2}$ being equal to 0.01 and 0.03 by default, respectively.

*Mean Aggregate Error (MAE)*

$$MAE=\frac{1}{N}\sum_{i=1}^{N} \left( y_{i}- \hat{y_{i}} \right)$$

With all terms being defined as in the MSE metric, above.

*Dice Similarity Coefficient (DSC)*

When measuring the pixel overlap between two segmented regions (e.g., segmentation on a ground truth image vs segmentation on a synthetic image), the Dice coefficient is given as the ratio of twice the intersection, divided by the sum of the union and the intersection, which in turn is proportional to the ratio of twice the true positives divided by the sum of twice the true positives plus the false negatives and the false positives:

$$DSC= \frac{2\times Intersection}{Union+Intersection}= \frac{2\times TP}{2\times TP+FN+FP}$$

*Accuracy*

$$Acc= \frac{TP+TN}{TP+TN+FP+FN}$$

Where TP, TN, FP, and FN stand for true positives, true negatives, false positives, and false negatives, respectively.

*Sensitivity*

$$Sens= \frac{TP}{TP+FN}$$

*Specificity*

$$Spec= \frac{TN}{TN+FP}$$

*Precision*

$$Prec= \frac{TP}{TP+FP}$$

*AUROC*

The area under the receiver operating characteristic curve is evaluated as the area under the cumulative distribution function of the true positive rate (TPR) against the false positive rate (FPR). The area is calculated by the trapezoidal sum rule for discrete values of TPR and FPR.

***A.2. Radiomics***

In our radiomic feature extraction mentioned in the preceding Materials and Methods section, we mentioned that we extracted only some original features. By “original” we mean unfiltered features. Specifically, we extracted shape features like elongation, flatness, major axis length, minor axis length, maximum 3D diameter, sphericity, and surface area. We also extracted first-order features such as first-order energy, first-order entropy, first-order kurtosis, first-order mean, and first-order skewness. Finally, we extracted selected features from the grey level co-occurrence matrix (GLCM) such as the GLCM-contrast, the GLCM-correlation, and the GLCM-inverse difference moment, selected features from the neighboring grey tone difference matrix (NGTDM) such as the NGTDM-coarseness, NGTDM-complexity, and NGTDM-strength. The definition of all features can be found in the literature from the IBSI^[1]^, and in the PyRadiomics online documentation^[2]^.
 *First-order Features*

First order features describe the distribution of voxel intensities within the image region defined by the segmentation mask through commonly used and basic metrics.

*Shape Features (3D)*

Shape features include descriptors of 3D size and shape of each segmentation mask and are independent of grey level intensity distribution in the ROI.

*Grey Level Co-occurrence Matrix Features (GLCM Features)*

A GLCM feature describes the second-order joint probability function of an image region constrained by the segmentation mask and represents the number of times the combination of grey levels i and j occur in two pixels in the image. PyRadiomics by default computes symmetrical GLCM features.

*Neighbouring Grey Tone Difference Matrix Features (NGTDM Features)*

A NGTDM feature quantifies the difference between a grey value and the average grey value of its neighbours within a certain distance. The sum of the absolute differences of grey level is stored in the matrix, and the computation is performed for all available grey tone values, and the total number of voxels within the segmentation mask.

1. Zwanenburg, A., Leger, S., Vallières, M., and Löck, S. (2016). Image biomarker standardisation initiative - feature definitions. In eprint arXiv:1612.07003
2. https://pyradiomics.readthedocs.io/en/latest/features.html#id3
